# Laboratory analysis and ray visualization of diffractive optics with enhanced intermediate vision

**DOI:** 10.1186/s12886-021-01958-8

**Published:** 2021-05-04

**Authors:** Hyeck-Soo Son, Grzegorz Łabuz, Ramin Khoramnia, Timur M. Yildirim, Gerd U. Auffarth

**Affiliations:** Department of Ophthalmology, The David J. Apple International Laboratory for Ocular Pathology and International Vision Correction Research Centre (IVCRC), University of Heidelberg, INF 400, 69120 Heidelberg, Germany

**Keywords:** Ray propagation, Optical quality, Extended-depth-of-focus, Multifocal IOL, Optical bench

## Abstract

**Background:**

To assess the optical behavior of a new diffractive intraocular lens (IOL) and compare its performance to that of an established extended-depth-of-focus (EDOF) IOL.

**Methods:**

This study assessed the Proming EDOF Multifocal AM2UX [Eyebright Medical Technology (Beijing) Co., Ltd., China] and the AT LARA 829MP [Carl Zeiss Meditec, Germany]. An experimental set-up with 0.01% fluorescein solution and monochromatic light (532 nm) was used to visualize the IOLs’ ray propagation. In addition, the optical quality of the IOLs was assessed by measuring the modulation transfer function (MTF) values at 50lp/mm and 3.0 and 4.5 mm apertures on the optical bench OptiSpheric® IOL PRO II [Trioptics GmbH, Germany].

**Results:**

The ray propagation of the two IOLs showed two distinct foci. Light intensity assessment revealed that both IOLs allocate more energy to primary than secondary focus. At 3.0 mm pupil, the MTF values at 50lp/mm for the primary focus were 0.39 and 0.37, and for the secondary focus, 0.29 and 0.26 for the AT LARA and Proming IOLs, respectively. At 4.5 mm pupil, the single-frequency MTF for the primary focus was 0.51 and 0.24 and for the secondary focus 0.21 and 0.15 for the AT LARA and Proming IOLs, respectively.

**Conclusions:**

When tested with an aberration-free model cornea under monochromatic conditions, the Proming behaved as a low-add bifocal lens; however, its properties did not differ much from the well-established AT LARA EDOF IOL. The AT LARA outperformed the Proming at low defocus (up to 2D), while the latter demonstrated better image quality in the 2-3D range.

## Introduction

With ever-increasing life expectancy, aging conditions such as cataract and presbyopia continue to pose a global health challenge causing considerable visual impairment in both low- and high-income countries [1]. While cataract leads to cloudy vision, presbyopia is not only associated with progressive loss of accommodation, but also with economic burden resulting from low productivity as patients fail to perform mundane tasks at near and intermediate distances [2]. As a result, there is a growing demand for solutions that can treat both cataract and presbyopia simultaneously.

Conventionally, cataract removal is followed by implantation of an intraocular lens (IOL) [3]. One can distinguish between two types of IOLs: a monofocal and a multifocal one. While monofocal lenses restore excellent visual acuity at far distance, they fail to provide a focal point at near distance [4, 5]. Multifocal IOLs, in contrast, can also relieve the presbyopic symptoms by enhancing vision at various distances [6].

Currently, there are different multifocal lenses available. While bifocal IOLs allow functional vision at far and near distances, trifocal IOLs can provide vision at intermediate distance additionally [6]. The recently introduced Extended-Depth-of-Focus (EDOF) IOLs have also become a popular option for patients who wish to be spectacle independent [7–14]. In contrast to bifocal or trifocal IOLs, EDOF IOLs are specifically designed to provide functional vision over an extended range of vision [7–14].

However, the EDOF term may be misplaced by IOL manufacturers to commercially label and characterize multifocal IOLs which have an increased depth of focus rather than a designated focus. As a result, the American Academy of Ophthalmology released a Task Force Consensus Statement to help define the minimum performance required to classify an IOL as an EDOF lens [15]: its monocular mean best-corrected distance visual acuity (BCDVA) should be non-inferior to that of a monofocal control, the distance-corrected intermediate visual acuity (DCIVA) should be superior to that of a monofocal control, and the monocular depth-of-focus should be at least 0.5 D greater than that of a monofocal control at 0.2 logMAR (20/32) [15].

Recently, a novel diffractive IOL was introduced to the market, the Proming EDOF Multifocal AM2UX, which purportedly has optical properties that qualify it as an EDOF lens. Therefore, the aim of this research was to assess and compare the optical performance of the Proming lens to that of the AT LARA IOL, a widely established EDOF lens [16–18], by measuring the through-focus (TF) modulation transfer function (MTF) values and using the ray-propagation imaging technique [19].

## Materials and methods

### Intraocular lenses

The following IOLs were assessed: the Proming EDoF Multifocal AM2UX [Eyebright Medical Technology (Beijing) Co., Ltd., China], and the AT LARA 829 MP [Carl Zeiss Meditec, Germany] IOLs.

The Proming AM2UX is a hydrophobic-acrylic lens with a refractive index of 1.48 and an Abbe number of 57. The IOL features an aspheric anterior surface and a diffraction grating on the posterior surface. According to the manufacturer, its aspheric-diffractive design provides a continuous range of vision from primary (far) to secondary (near) focus.

The AT LARA is a refractive-diffractive IOL manufactured from hydrophilic (25%) acrylic material with 1.46 refractive index and 56.5 Abbe number. The lens has an aspheric design that is “aberration neutral,” and it also features a chromatic-aberration correction [16, 17].

Five samples from each IOL model were tested, each with the same refractive power of 20.0D.

### Optical quality evaluation

The OptiSpheric IOL Pro II (Trioptics GmbH, Germany) instrument was used to measure the optical quality of the IOLs in accordance with the ISO 11979 standard [20]. Briefly, the optical bench consists of a light source, a reticle, a collimator, an eye model, a microscope, and a CCD camera. The reticle was illuminated by a collimator and imaged by the IOL under test onto the CCD camera. This set-up was used to measure the effective focal length (EFL) and the MTF.

The EFL was measured in monochromatic (green) light with the magnification method described in the ISO standard [20]. The power (P) was calculated from the EFL, and the measurements were carried out without a model cornea [20]. An aperture of 3.0 mm and a square reticle were used.

The MTF was calculated via the Fourier transform of the line spread function imaged by the studied lens [21]. The MTF was measured in monochromatic (546 nm) light at 3.0 and 4.5 mm apertures. The measurements were performed with an aberration-neutral model cornea, which was chosen to objectively compare the IOLs under standardized conditions guided by the industry standard for testing IOLs. The MTF was compared at a single spatial frequency of 50lp/mm, which was also the cut-off for the MTF Strehl ratio calculated as the area under the IOL’s MTF normalized by the area under a diffraction-limited MTF [21]. The TF MTF was assessed at 50lp/mm with a defocus range of − 0.5 to 3.5D (at the IOL plane), starting from the (best) far lens focus. Additionally, images of the United States Air Force (USAF) target were recorded for the same defocus range in 0.5D step.

### Ray propagation visualization

The ray propagation of the IOLs was visualized using the same technique as described in a previous study [19]. In short, the study IOL is placed in a lens holder with a 3 mm opening that is submerged in a water tank (1 L) with a 0.01% fluorescein solution. A monochromatic green laser light (532 nm) is then projected through both a model cornea and the IOL, and the ray propagation is visualized and captured with a digital camera mounted on a surgical microscope placed above the tank. The image-processing software ImageJ, provided by the US National Institute of Health, was used to determine the pixel intensity of the visualized rays along the optical axes.

## Results

### Power measurements

The mean nominal power of the Proming and AT LARA IOLs were 20.29 ± 0.09D and 20.04 ± 0.20D, respectively. The Proming’s add power was 2.47 ± 0.02D, which was higher than 1.87 ± 0.01D found in the AT LARA.

### Optical performance measurements

**Fig. 1:**
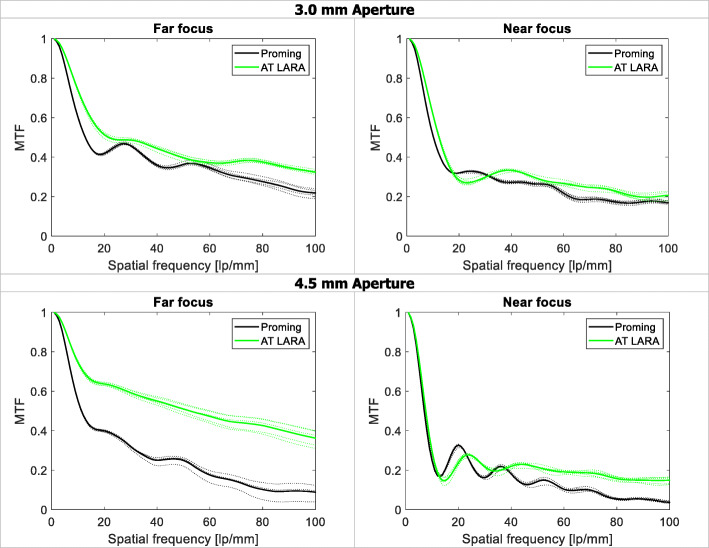
The modulation transfer function (MTF) of the Proming and AT LARA IOLs measured at 3.0 and 4.5 mm pupil sizes. The dashed line shows the results of individual IOLs; the solid line shows the average value

Figure 1 shows the MTF curves of the two IOLs measured at the primary and secondary foci. The positions of the secondary focus depended on the IOL model’s add power. Tables 1 and 2 summarize the MTF values and Strehl ratio results at the primary and secondary foci for both 3.0 and 4.5 mm apertures. At 3.0 mm pupil size (Table 1), the AT LARA showed slightly higher MTF and Strehl ratio values for both primary and secondary foci than the Proming. At 4.5 mm pupil size (Table 2), the AT LARA had a 2- and 1.5-fold higher MTF (at 50 lp/mm) and Strehl ratio results, respectively, than those of the Proming lens for primary focus. For secondary focus, the Proming’s image-quality metrics were worse by 0.06 (MTF) and 0.03 (Strehl ratio) compared to those measured in the AT LARA.

**Table 1:** Modulation transfer function (MTF) values of the lenses at 50lp/mm and Strehl Ratio results for the primary and secondary foci (3.0 mm pupil)

		MTF @ 50lp/mm		Strehl Ratio	
		Mean	SD	Mean	SD
*Primary Focus*	**Proming**	0.37	0.01	0.54	0.00
	**AT LARA**	0.39	0.01	0.60	0.00
*Secondary Focus*	**Proming**	0.26	0.01	0.44	0.00
	**AT LARA**	0.29	0.01	0.48	0.00

*SD = standard deviation*

**Table 2:** Modulation transfer function (MTF) values of the lenses at 50lp/mm and Strehl Ratio results for the primary and secondary foci (4.5 mm pupil)

		MTF @ 50lp/mm		Strehl Ratio	
		Mean	SD	Mean	SD
*Primary Focus*	**Proming**	0.24	0.02	0.44	0.00
	**AT LARA**	0.51	0.02	0.67	0.00
*Secondary Focus*	**Proming**	0.15	0.02	0.30	0.00
	**AT LARA**	0.21	0.01	0.33	0.00

*SD = standard deviation*

### Defocus range analysis

**Fig. 2:**
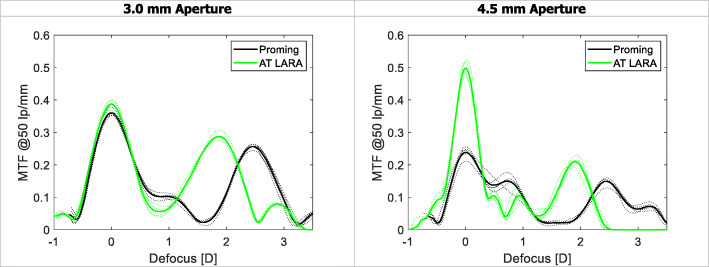
The through-focus modulation transfer function (MTF) of the two lenses at 50lp/mm for 3.0 and 4.5 mm apertures. The dashed line shows the results of individual IOLs, the solid line shows the average value

**Fig. 3:**
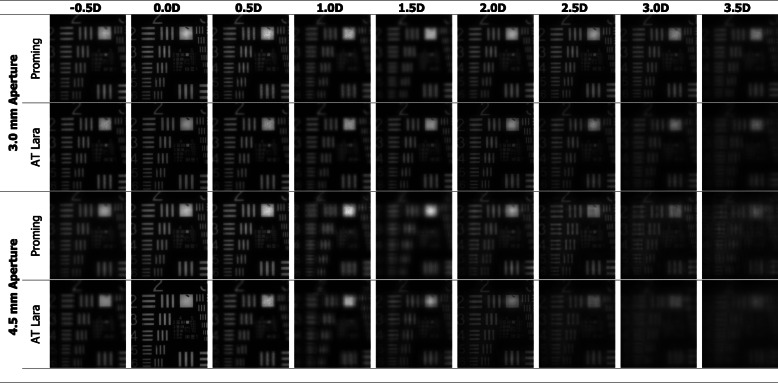
USAF target images recorded at a defocus range of − 0.5D to 3.5D and apertures 3.0 mm and 4.5 mm

The results of the TF MTF measurements at a spatial frequency of 50lp/mm are shown in Fig. 2. The USAF target images are presented in Fig. 3. The TF analysis performed at the 3.0 mm aperture demonstrated that both the Proming and AT LARA IOLs allocate more light energy to the primary than secondary focus. The two IOLs revealed a clear separation of TF MTF peaks corresponding to the two main foci. The difference in the secondary-focus position resulted from different add powers of the studied lens models. The AT LARA lens showed slightly higher secondary-peak at its intended add power than the Proming lens. At the 4.5 mm aperture, the AT LARA exhibited an excellent TF MTF value for zero defocus. In contrast, the Proming displayed lower, but slightly extended primary-peak. The secondary-peak of the AT LARA was also higher than that of the Proming lens at this pupil size. The USAF target images (Fig. 3) confirmed the TF MTF results qualitatively, with the Proming IOL providing a wide range of vision up to 3D; yet, the images taken at 1D and 1.5D appear blurred. Although the AT LARA’s imaging quality seems to be superior at this range, it becomes inferior for more than 2.5D of defocus.

### Ray-propagation visualization

**Fig. 4:**
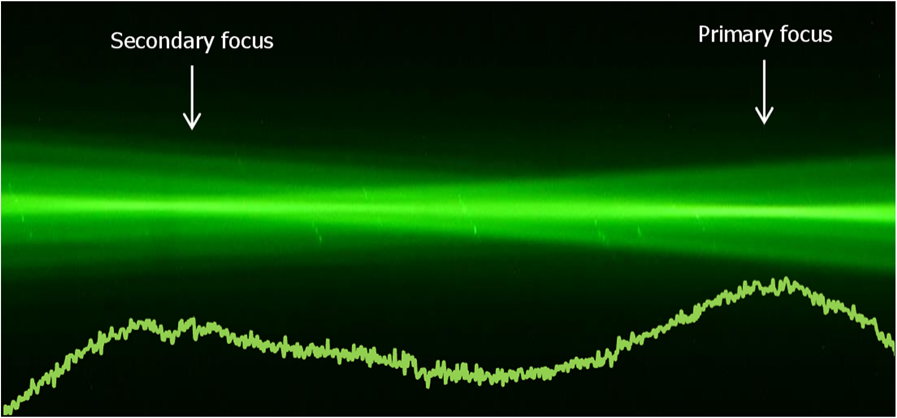
Light-pathways visualization and the light intensity profile (solid line) of the Proming Multifocal lens

**Fig. 5:**
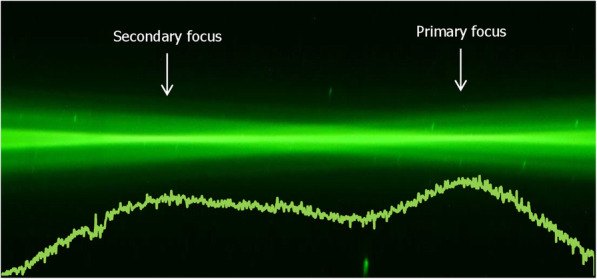
Light-pathways visualization and the light intensity profile (solid line) of the AT LARA lens

**Fig. 6:**
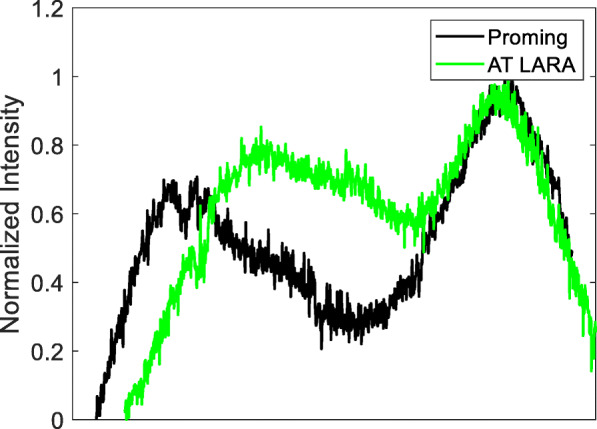
The comparison of the light-intensity profiles of the studied lenses. The dashed line corresponds to the position of the primary (far) focus

Figures 4 and 5 show the green-laser light propagation (from left to right) projected by the Proming (Fig. 4) and AT LARA (Fig. 5) IOLs. The light intensity profile is presented in each figure under the ray bundles and directly compared in Fig. 6.

Both IOLs demonstrated two distinct foci that correspond to the nominal and add powers, respectively. The Proming and the AT LARA showed a comparable light distribution pattern with more light energy directed to the primary focus. The light intensity assessment of the IOLs (Fig. 6) appears to agree with the TF MTF measurements (Fig. 2).

## Discussion

In this experimental study, we showed that the Proming IOL provides good MTF performance, which is close to that of its counterpart lens for a small (3.0 mm) aperture size. However, at the increased aperture (4.5 mm), differences became apparent between the studied designs. Furthermore, the two IOL models differ in the defocus (visual) range in which they provide satisfactory image quality. To the best of the authors’ knowledge, this is the first laboratory study characterizing the optical performance of the Proming IOL.

The Proming IOL’s MTF values at the primary focus were only minimally lower than those of the AT LARA for the 3.0 mm pupil (Fig. 2**,** Table 1). However, when the aperture size increased, the AT LARA outperformed the Proming lens (Fig. 2**,** Table 2), which results from differences in the amount of spherical aberration induced by each model. This impact of spherical aberration on image and visual quality has been reported by many researchers [22–24]. Note that the AT LARA features an aberration-neutral design. As we used the model cornea that is also aberration-neutral, the AT LARA’s performance, ideally, would not be affected by spherical-aberration, resulting in excellent image quality. By contrast, if an aberration-correcting design were studied with an aberration-free cornea, its image quality may be degraded due to increased negative spherical-aberration. One may wonder whether the match of the model cornea and the Proming IOL caused its decreased optical performance at the 4.5 mm aperture. In this study, we analyzed the optical quality using a model cornea that complements the design of the AT LARA. The Proming’s manufacturer has not disclosed the level of spherical aberration. Thus, we could not match a model cornea for its asphericity, nor the reasons for Proming’s poor imaging quality at scotopic pupil could be discussed.

To this date, two laboratory studies characterized the optical performance of the AT LARA IOL [16, 17]. We previously measured the lens using an aberrated model cornea (+ 0.28 μm) and also observed good image quality from its primary and secondary foci at 3 mm, but it was reduced at 4.5 mm [17]. Furthermore, we found that at primary focus, the AT LARA showed slight deterioration in its optical quality when measured with polychromatic than with monochromatic light. Yet, the difference was less pronounced than in a refractive EDOF lens, as the AT LARA employs chromatic aberration correction [17]. In another study, we evaluated the influence of longitudinal chromatic aberration (LCA) on the polychromatic optical quality of different multifocal lenses. We found that the AT LARA lens is able to compensate for the chromatic aberration better than other diffractive IOLs, with LCA of 0.78D at the primary focus. A value that is lower than that of an aphakic model eye (1.04D) [16]. At the secondary focus, the correction was more effective inducing only 0.21D of residual LCA, which led to a minimal change of the AT LARA’s optical quality compared to a single-wavelength measurement. The polychromatic MTF at 50 lp/mm was 0.30 and 0.23 at the far and intermediate focus, respectively [17]. While we used monochromatic conditions in this study, our results may correspond with those obtained in polychromatic light due to the low chromatic effects of the AT LARA.

The AT LARA has also been studied clinically [9]. In a recent paper, the visual outcomes of 11 patients with the AT LARA IOL implanted bilaterally were evaluated [9]. The authors found good binocular corrected distance visual acuity of − 0.01logMAR and distance-corrected near visual acuity of 0.33logMAR (at 40 cm) 3-months postoperatively [9]. It was reported that the AT LARA IOL demonstrated better performance at intermediate than at near range, with binocular distance-corrected intermediate visual acuity values of − 0.07, 0.04, and 0.07logMAR at 90, 80, and 60 cm, respectively [9], which are in conformity with our results.

The TF MTF and the ray-visualization analysis displayed two distinct foci with the Proming IOL having the secondary MTF peak recorded at approximately 2.5D (Figs. 2, 3, and 6). Interestingly, Fig. 2 shows an extended far focus up to 1.5D, but this did not result in improved image quality at a 1–1.5D range, as one can see from the USAF resolution-chart photographs (Fig. 3). At 0.5D, however, this (far) TF MTF elongation may have led to an improvement in Proming IOL’s performance as its image quality was noticeably better than that of the AT LARA, particularly at 4.5 mm (Fig. 3). However, whether patients can perceive this as an EDOF effect remains to be elucidated in a clinical study. The AT LARA also had two distinct peaks in the TF MTF scan, with the secondary peak positioned at about 1.87D (Figs. 2, 3, and 6). A relatively small separation of the AT LARA’s foci resulted in better optical quality than the Proming at the intermediate range (Fig. 3). On the other hand, the Proming provided better image quality at near (Fig. 3), which may improve patients’ reading performance.

The power measurement results indicate that the studied lenses were correctly labeled for their nominal power as the reported values were within an ISO tolerance limit of ±0.4D [20]. Furthermore, the low standard deviation of the measured nominal power and minimal variability of the optical quality parameters suggest there is good reliability in the IOL’s manufacturing process.

In conclusion, the new Proming IOL showed good image quality and behaved as a low-add bifocal lens when tested with an aberration-free model cornea under monochromatic conditions, similar to other commercially established EDOF lenses [17]. However, whether it meets the American Academy of Ophthalmology requirements for EDOF lenses has to be addressed in a clinical study. At the far focus, the MTF was as good as that of the AT LARA in the presence of low spherical aberration at 3.0 mm pupil. Although the AT LARA provided better MTF performance than the Proming IOL at low defocus (up to 2D), the latter demonstrated better image quality in the 2-3D range. The ray visualization and the TF MTF data confirmed an enhanced range of vision produced by the studied IOLs.

## Data Availability

Authors can confirm that all relevant data are included in the article. The datasets used for analysis are available from the corresponding author on reasonable request.
